# Effects of bile acids on production performance, serum biochemistry, lipid metabolism, and intestinal morphology in broilers

**DOI:** 10.3389/fvets.2025.1584544

**Published:** 2025-04-30

**Authors:** Wenjing Liu, Xinan Li, Yunlong Zhang, Tao Li, Yuqiang Wei, Xuejun Yuan, Yang Li, Weiren Yang, Ning Jiao

**Affiliations:** ^1^Key Laboratory of Efficient Utilization of Non-Grain Feed Resources (Co-construction by Ministry and Province), Ministry of Agriculture and Rural Affairs, College of Animal Science and Technology, Shandong Agricultural University, Tai’an, China; ^2^Daiyue District Livestock and Veterinary Development Service Center, Tai’an, China; ^3^Linyi Zhengneng Biotechnology Co., Ltd., Linyi, China; ^4^College of Life Sciences, Shandong Agricultural University, Tai’an, China

**Keywords:** bile acids, slaughter characteristics, serum enzyme, lipid metabolism, intestinal morphology

## Abstract

The objective of the present study was to evaluate the effects of exogenous bile acids (BAs) on production performance, serum biochemistry, lipid metabolism, and intestinal morphology in broilers. A total of 20,000 one-day-old Arbor Acres broilers were randomly assigned to two treatment groups, with eight replicates of 1,250 chicks each, for a duration of 42 days. The control group broilers were provided with normal drinking water, while the treatment group broilers were provided with drinking water supplemented with 100 mL of BAs per ton. The results showed that BAs supplementation significantly decreased abdominal fat yield (*p* < 0.05), while having no significant effect on other slaughter characteristics (*p* > 0.05). In addition, BAs supplementation significantly decreased serum acrylic aminotransferase concentrations (*p* < 0.05). Moreover, BAs supplementation also significantly decreased the mRNA expression of lipogenesis-related genes (*p* < 0.05) and increased the mRNA expression of lipolysis-related genes in the liver (*p* < 0.05). Furthermore, BAs supplementation significantly improved jejunal morphology by increasing jejunum villus height (VH) (*p* < 0.05). In summary, BAs supplementation improved production performance, serum enzyme activity, lipid metabolism, and intestinal morphology in broilers, providing a theoretical basis for the application of BAs in broiler production.

## 1 Introduction

In recent years, high-fat diets have widely been used to increase dietary energy levels, with the goal of improving growth performance and feed conversion efficiency in the broiler industry (1). However, excessive dietary fat could lead to increased accumulation of carcass fat in the abdomen, induce metabolic disorders, and reduce both feed efficiency and carcass yield (2, 3). In addition, limited emulsifying capacity restricts lipid utilization and optimal growth performance in broilers (4). Therefore, various emulsifiers have been used in the broiler industry to optimize the digestion and absorption of dietary lipids, improving dietary energy utilization and maintaining efficient productivity (5, 6). In addition, emulsifiers could enhance lipid digestion by reducing the surface tension of lipid droplets and allowing physical agitation in the gastrointestinal tract to break them down into smaller particles (7). Studies have reported that emulsifiers could enhance the fat utilization efficiency of broilers by inhibiting hepatic fatty acid synthesis and promoting hepatic triglyceride (TG) synthesis and transport capacity (8).

Bile acids (BAs) are unique and significant organic components of bile, acting as natural endogenous emulsifiers. BAs are essential for the absorption of lipids and fat-soluble vitamins. They maintain the dynamic balance between cholesterol synthesis and excretion in the liver while promoting the absorption and transport of lipids, vitamins, and nutrients in the intestines (9). Studies have reported that exogenous BAs are widely used in poultry production due to the low efficiency of fat digestion and utilization in poultry (10, 11). It has been reported that dietary supplementation with BAs improves the average daily gain, feed conversion ratio, and antioxidant capacity in heat-stressed broilers (12). BAs also effectively improved the growth performance and carcass traits in broilers by increasing the activity of lipoproteins and lipases in the duodenum (13). In addition, another study showed that dietary BAs improved growth performance and alleviated fatty liver by modulating the intestinal flora of broilers (14). Research has shown that dietary supplementation with bile salts effectively regulates the mRNA expression of lipid metabolism genes and plays an essential role in improving fat deposition in yellow feather broilers (15). However, studies on lipid metabolism of BAs in broilers are limited. Therefore, this study aimed to evaluate the effects of BAs as exogenous emulsifiers on slaughter performance, serum biochemistry, intestinal morphology, and the mRNA expression of lipid metabolism-related genes in broilers.

## 2 Materials and methods

### 2.1 Preparation of bile acids

BA additives were sourced from Shandong Zhongjing Biotechnology Company Limited (Shandong, China). The composition included 3.05% chenodeoxycholic acid, 6.59% seal bile acid, 0.24% hyocholic acid, 0.49% hyodeoxycholic acid, 11.16% cholic acid, and a moisture content of ≤10.00%.

### 2.2 Animals, treatments, and management

The Animal Care and Use Committee of Shandong Agricultural University (Beijing, China) approved the animal experiment (SDAUA-2021-081). A total of 20,000 one-day-old Arbor Acres broilers were randomly divided into two treatment groups, with eight replicates for each treatment group consisting of 1,250 broilers per replicate. The 42-day experiment was conducted in two phases: first phase (days 0–21) and growth phase (days 21–42). The control group broilers were provided with normal drinking water (CON), while the treatment group broilers were provided with drinking water supplemented with 100 mL of BAs per ton (BAs). The broilers were caged and fed a pelleted diet. The broilers had unrestricted access to feed and water throughout the experiment. The diet was formulated to meet or surpass the nutritional requirements for broiler chickens, as calculated according to The National Research Council (NRC, 1994). The ingredients and chemical composition of the basal diet are shown in Table 1.

**Table 1:** Ingredients and nutrient levels of the basal diets (%, air-dried basis).

Items	1 ~ 21 d of age	22 ~ 42 d of age
Ingredients		
Corn	54.10	58.43
Soybean meal	24.20	20.20
Wheat flour	8.22	8.00
Corn protein powder	3.10	2.00
Cottonseed meal	4.00	3.80
Feather meal	1.00	1.27
CaHPO_4_	1.90	1.75
Pulverized limestone	1.00	1.13
Soybean oil	0.00	1.00
Lysine	0.24	0.26
DL-methionine	0.18	0.09
L-threonine	0.06	0.07
Premix^a^	2.00	2.00
Total	100	100
Nutrient levels		
ME (MJ/kg)	12.93	13.12
Crude protein	21.00	19.00
Calcium	0.90	0.90
Total phosphorus	0.43	0.40
Lysine	1.10	1.00
Methionine	0.50	0.38
Sulfur-containing amino acids	0.86	0.72
Threonine	0.80	0.74

a The premix was provided per kg of the diet as follows: VA, 9,050 IU; VD_3_, 1,950 IU; VE, 26 mg; VK_3_, 5.0 mg; VB_1_, 2.6 mg; VB_2_, 8.0 mg; VB_6_, 3.0 mg; VB_12_, 0.02 mg; chloride, 500 mg; calcium pantothenic, 15 mg; niacin, 35 mg; biotin, 0.20 mg; folic acid, 1.2 mg; Mn (as MnSO_4_·H_2_O), 60 mg; Fe (as FeSO_4_·H_2_O), 80 mg; Zn (as ZnSO_4_·H_2_O), 60 mg; Cu (as CuSO_4_·5H_2_O), 8.5 mg; I (as KIO_3_), 0.27 mg; and Se (as Na_2_SeO_3_), 0.20 mg.

### 2.3 Slaughter and sample collection

At the end of the experiment, one healthy broiler from each replicate, which visually approximated the average weight, was selected for blood sampling and slaughtering. Blood samples were collected from the wing vein under fasting conditions using a blood collection needle, and 5 mL of blood was collected using a vacuum procoagulant tube. Serum was obtained by centrifuging the blood at 3,500 × g for 15 min and stored at −20°C until further analysis.

After blood sampling, the broilers were weighed and slaughtered. The giblets were removed to obtain the semi-eviscerated weight. The subsequent removal of the internal organs from the semi-eviscerated carcass resulted in the eviscerated carcass weight. Abdominal fat, extra-muscular stomach fat, breast muscle, and leg muscle were collected and weighed. The slaughtering rate, eviscerated rate, semi-eviscerated rate, abdominal fat yield, breast muscle rate, and leg muscle rate were assessed following the Nomenclature and Metric Statistical Methods of Poultry Production Performance (NY/T 823-2020).

Liver tissue samples from the broilers were quickly removed and collected, snap-frozen in liquid nitrogen, and stored at −80°C. Other portions of the liver and the middle part of the jejunum were fixed in a 4% paraformaldehyde solution for morphological analysis.

$$\begin{array}{l} \mathrm{Slaughtering Rate}\;\left(\%\right)=\;\\ \;\mathrm{Slaughtered Body Weight}/\mathrm{Live Body Weight}\times 100\%\text{.} \end{array}$$

$$\begin{array}{l} \mathrm{Eviscerated Rate}\;\left(\%\right)=\;\\ \;\mathrm{Eviscerated Carcass Weight}/\mathrm{Live Body Weight}\times 100\%\text{.} \end{array}$$

$$\begin{array}{l} \mathrm{Semi}-\mathrm{Eviscerated Rate}\;\left(\%\right)=\;\\ \;\mathrm{Semi}-\mathrm{Eviscerated Carcass Weight}/\mathrm{Live Body Weight}\times 100\%\text{.} \end{array}$$

$$\begin{array}{l} \mathrm{Breast Muscle Rate}\;\left(\%\right)=\;\\ \;\mathrm{Breast Muscle Weight}/\mathrm{Eviscerated Carcass Weight}\times 100\%\text{.} \end{array}$$

$$\begin{array}{l} \mathrm{Leg}\;\mathrm{Muscle Rate}\;\left(\%\right)=\;\\ \;\mathrm{Leg}\;\mathrm{Muscle Weight}/\mathrm{Eviscerated Carcass Weight}\times 100\%\text{.} \end{array}$$

$$\begin{array}{l} \mathrm{Abdominal}\;\mathrm{Fat}\;\mathrm{Rate}\;\left(\%\right)=\;\\ \;\mathrm{Abdominal}\;\mathrm{Fat}\;\mathrm{Weight}/\left(\begin{array}{l} \mathrm{Eviscerated Carcass Weight}\\ +\;\mathrm{Abdominal Fat Weight} \end{array}\right)\times 100\%\text{.} \end{array}$$

### 2.4 Serum biochemical indexes

The levels of serum enzymes, such as alkaline phosphatase (ALP), aspartate aminotransferase (AST), and aspartate transaminases (ALT), and the concentrations of metabolites, including total cholesterol (TC), triglycerides (TG), total protein (TP), glucose (GLU), blood urea nitrogen (BUN), high-density lipoprotein (HDL), low-density lipoprotein (LDL), and albumin (ALB), were measured using a COBUS MIRA Plus automatic biochemical analyzer (Roche Diagnostic System Inc., United States), following standard operating procedures.

### 2.5 Determination of the liver fat content and antioxidants parameter

The liver tissue samples were pulverized and broken, homogenized, and centrifuged with saline at a mass-to-volume ratio of 1:9. The supernatant was collected for measurement. The liver fat content, including triglyceride (TG) and total cholesterol (TC), and the antioxidants parameter of malondialdehyde (MDA) in the liver tissue were measured using an ELx800 enzyme labeling instrument (BioTek) with commercial kits (Nanjing Jiancheng Bio-engineering Institute, Nanjing, China), following the manufacturer’s instructions.

### 2.6 Morphological observation

At the end of the experimental period, liver and jejunum samples were collected for morphological assessment. Specifically, the liver and jejunum tissues fixed in paraformaldehyde were removed and trimmed. The tissues were quickly sliced vertically to a thickness of approximately 1 cm. They were placed into labeled embedding cassettes and rinsed with a slow stream of tap water for 48 h to remove the fixative. Then, the rinsed blocks were placed sequentially into ethanol solutions of 70, 80, 85, 90, 95, 100, and 100% for 2 h to remove water. Afterward, each tissue block was immersed in xylene until no oil residue was visible on the surface of the tissue. Subsequently, the blocks were transferred to liquid paraffin wax maintained at 60°C and left to incubate for 12 h. Following this, the blocks were infused with the liquid wax for embedding and sliced into 6 μm-thick sections using a microtome. After cutting, the sections were placed in a constant-temperature oven at 37°C for 1–2 days until completely dried. They were then stained with hematoxylin–eosin, covered with a coverslip, and dried again. The samples were examined using a Nikon Elipse 80i microscope (Nikon, Tokyo, Japan), and the pictures were captured using a DP25 digital camera. In addition, villus height (VH) and crypt depth (CD) were measured from the digital pictures, and the ratio of villus height to crypt depth was subsequently calculated.

### 2.7 Quantitative real-time PCR analysis

The tissue mRNA expression levels of lipid metabolism genes [hormone-sensitive lipase (*HSL*), stearoyl coenzyme A desaturase (*SCD*), lipoprotein lipase (*LPL*), fatty acid synthase (*FAS*), and fatty acid-binding protein 4 (*FABP4*)] were accessed using real-time fluorescence quantitative polymerase chain reaction (qPCR). Total RNA was extracted from the liver using the AG RNAex Pro Reagent (Accurate Biology Co., Ltd., Changsha, China) and quantified with a UV spectrophotometer at 260 and 280 nm absorbance ratios. Meanwhile, RNA integrity was verified via agarose gel electrophoresis using the GelDoc Go Gel imaging analysis system (Bio-Rad, United States). Then, Evo M-MLV Reverse Transcription Kit AG11706 (Accurate Biology Co., Ltd., Changsha, China) was used to remove genomic DNA and synthesize cDNA. Then, quantitative real-time polymerase chain reaction (qRT-PCR) was performed as described in a previous study (16). β-actin was used as the internal reference gene, and the 2^−△△Ct^ method was used to calculate the relative mRNA expression of the target genes. The primer sequences are listed in Supplementary Table S1.

### 2.8 Statistical analysis

All data were statistically analyzed using the general linear model (GLM) in SAS (version 9.4, SAS Institute, Cary, NC, United States), and differences between the treatments were compared using Student–Newman–Keuls tests. The results were presented as means ± SEMs. All statements of significance were based on a probability of a *p*-value < 0.05. Figures were created using GraphPad Prism (version 8, La Jolla, CA, United States).

## 3 Results

### 3.1 Production performance

**Figure 1:**
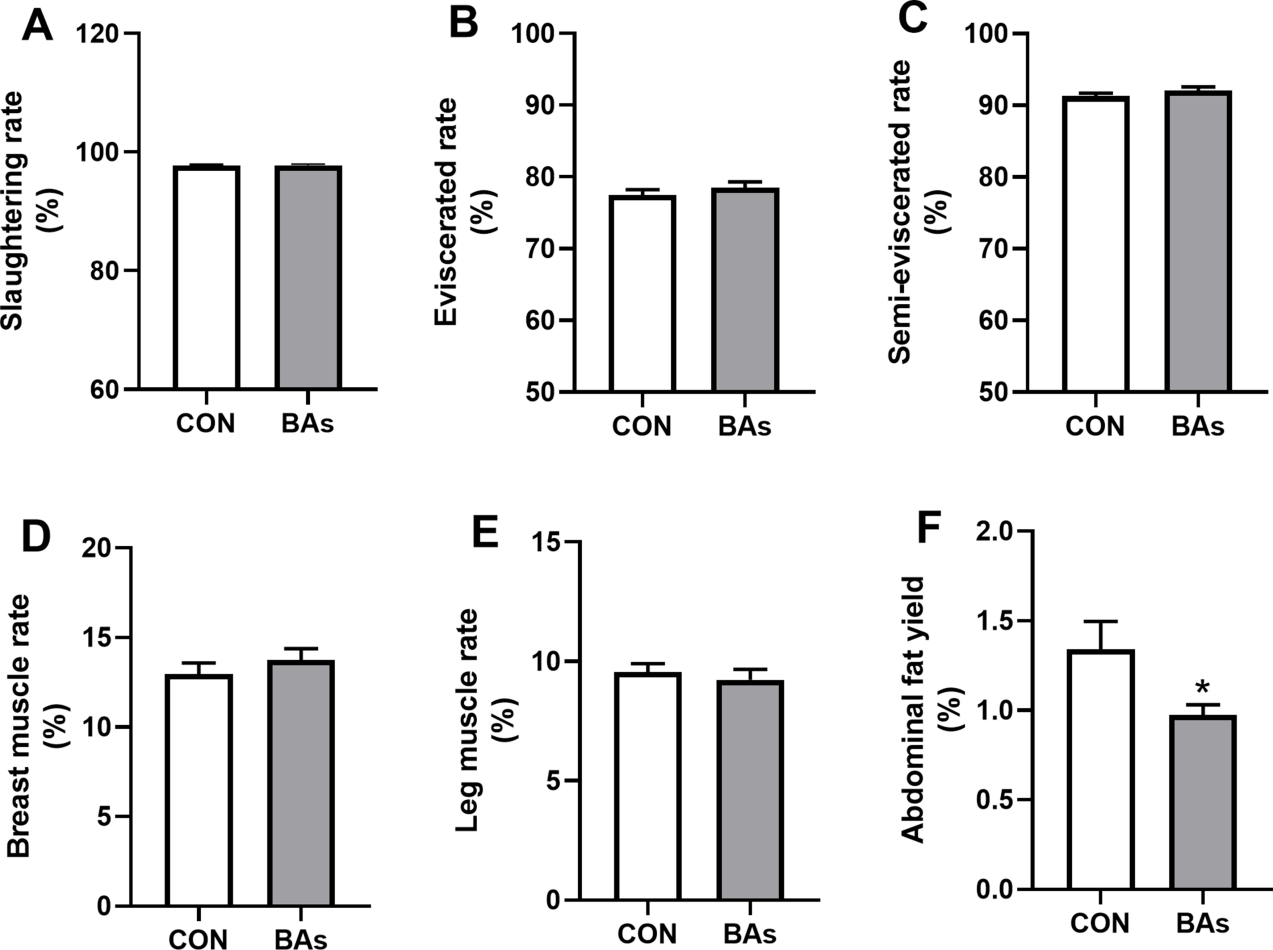
Effects of BAs supplementation on production performance in the broilers. **(A)** Slaughtering rate. **(B)** Eviscerated rate. **(C)** Semi-eviscerated rate. **(D)** Breast muscle rate. **(E)** Leg muscle rate. **(F)** Abdominal fat yield. The results were expressed as means ± SEMs, *n* = 8. *p-value < 0.05 compared to the CON group.

Figure 1 shows the effects of BAs on production performance in the broilers. Compared to the CON group, BAs significantly decreased the abdominal fat yield in broilers by 20.9% (*p* < 0.05). However, no significant differences were observed in the slaughtering rate, eviscerated rate, semi-eviscerated rate, breast muscle rate, and leg muscle rate between the CON and BAs groups (*p* > 0.05).

### 3.2 Serum biochemistry

**Figure 2:**
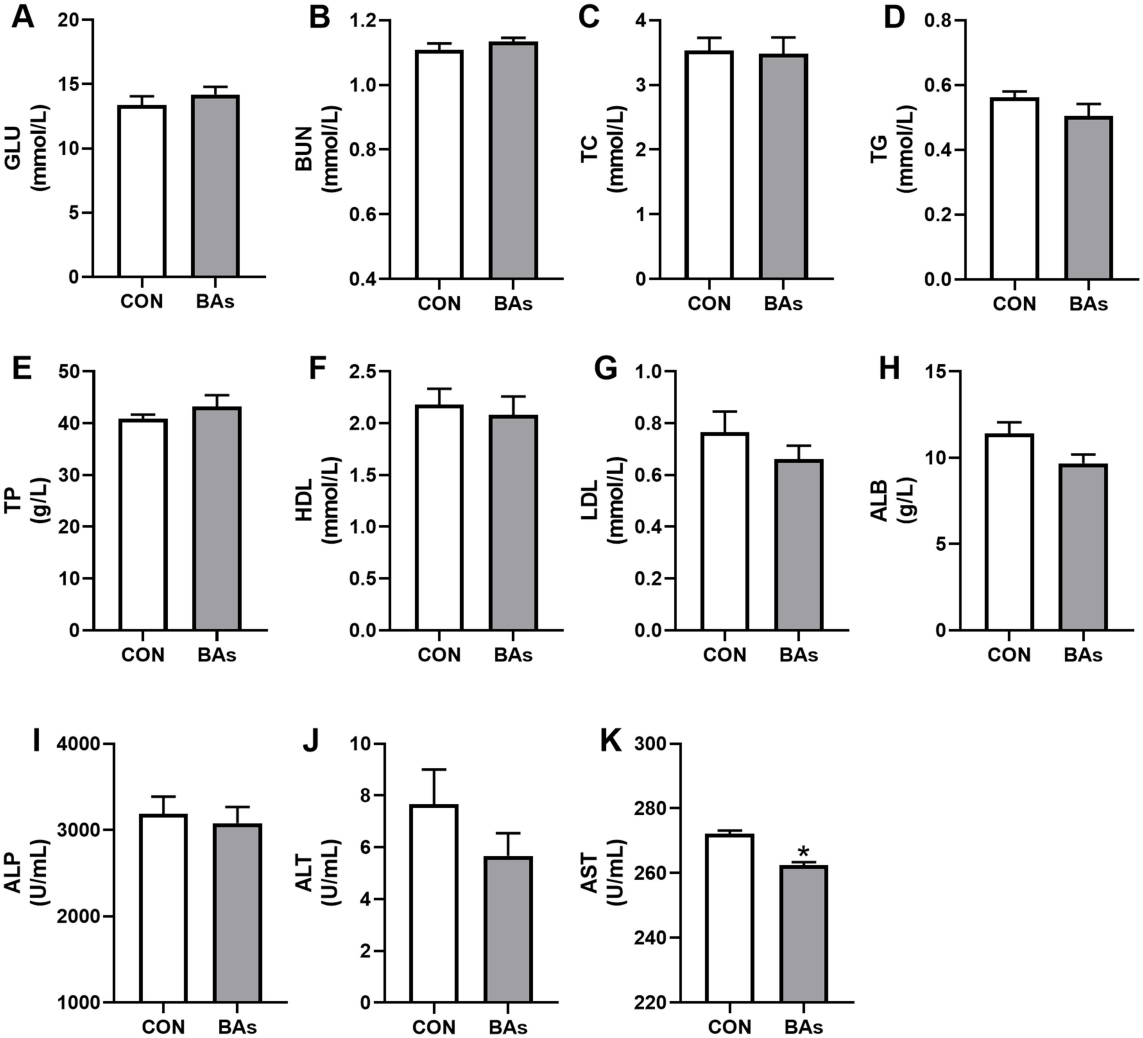
Effects of BAs supplementation on serum biochemistry in the broilers. **(A–H)** Serum levels of the metabolites, including glucose (GLU) **(A)**, blood urea nitrogen (BUN) **(B)**, total cholesterol (TC) **(C)**, triglycerides (TG) **(D)**, total protein (TP) **(E)**, high-density lipoprotein (HDL) **(F)**, low-density lipoprotein (LDL) **(G)**, and albumin (ALB) **(H)**. **(I–K)** Serum activity of alkaline phosphatase (ALP) **(I)**, aspartate transaminase (ALT) **(J)**, and acrylic aminotransferase (AST) **(K)**. The results were expressed as means ± SEMs, *n* = 8. *p-value < 0.05 compared to the CON group.

As shown in Figure 2, compared to the CON group, the enzyme activity of AST in the BAs group was significantly decreased by 3.6% (*p* < 0.05). However, there were no significant differences in the enzyme activity and serum concentrations of ALP, ALT, GLU, BUN, TC, TG, TP, HDL, LDL, and ALB between the two treatments (*p* > 0.05).

### 3.3 Liver fat content and antioxidant parameter

**Figure 3:**
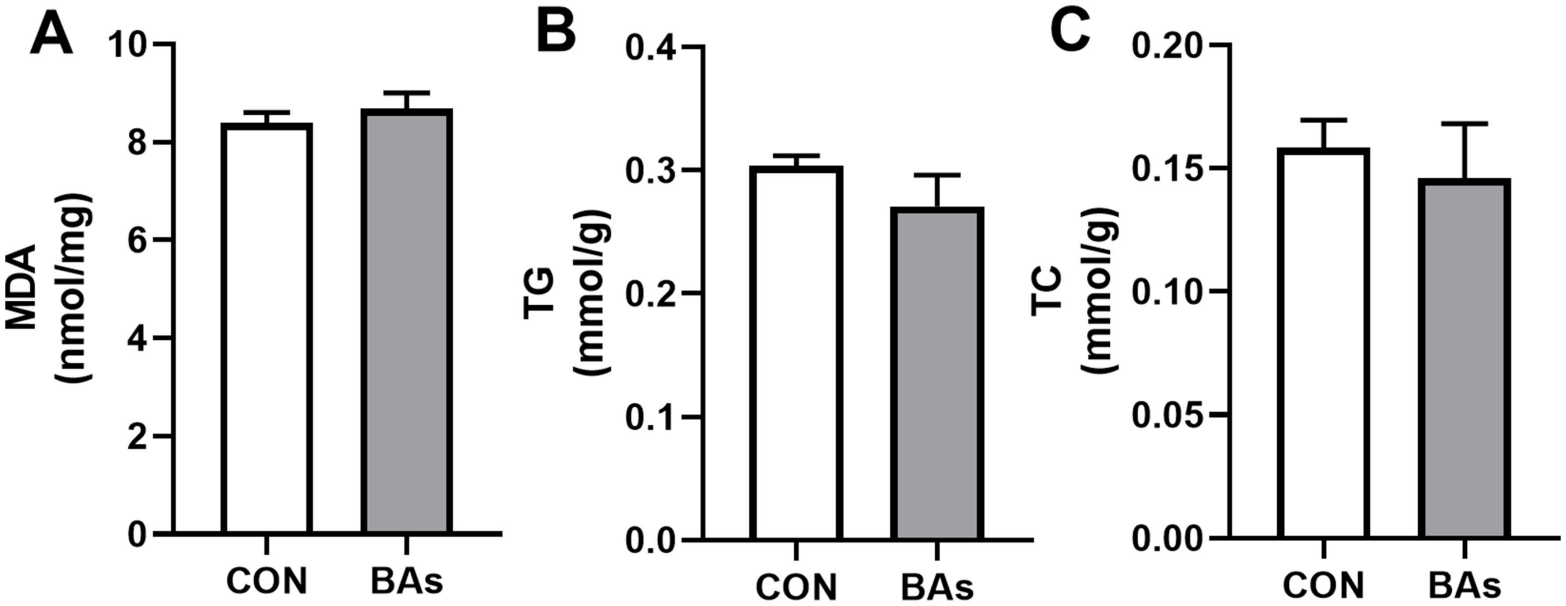
Effects of BAs supplementation on the liver fat content and antioxidant-related parameter in the broilers. **(A)** Malondialdehyde (MDA) content. **(B)** Triglyceride (TG) content. **(C)** Cholesterol (TC) content. The results were expressed as means ± SEMs, *n* = 8. *p-value < 0.05 compared to the CON group.

The liver fat content (TG and TC) and antioxidant-related parameter (MDA) levels are shown in Figure 3. Compared to the CON group, the addition of BAs had no significant effects on TG, TC, and MDA levels (*p* > 0.05).

### 3.4 mRNA expression

**Figure 4:**
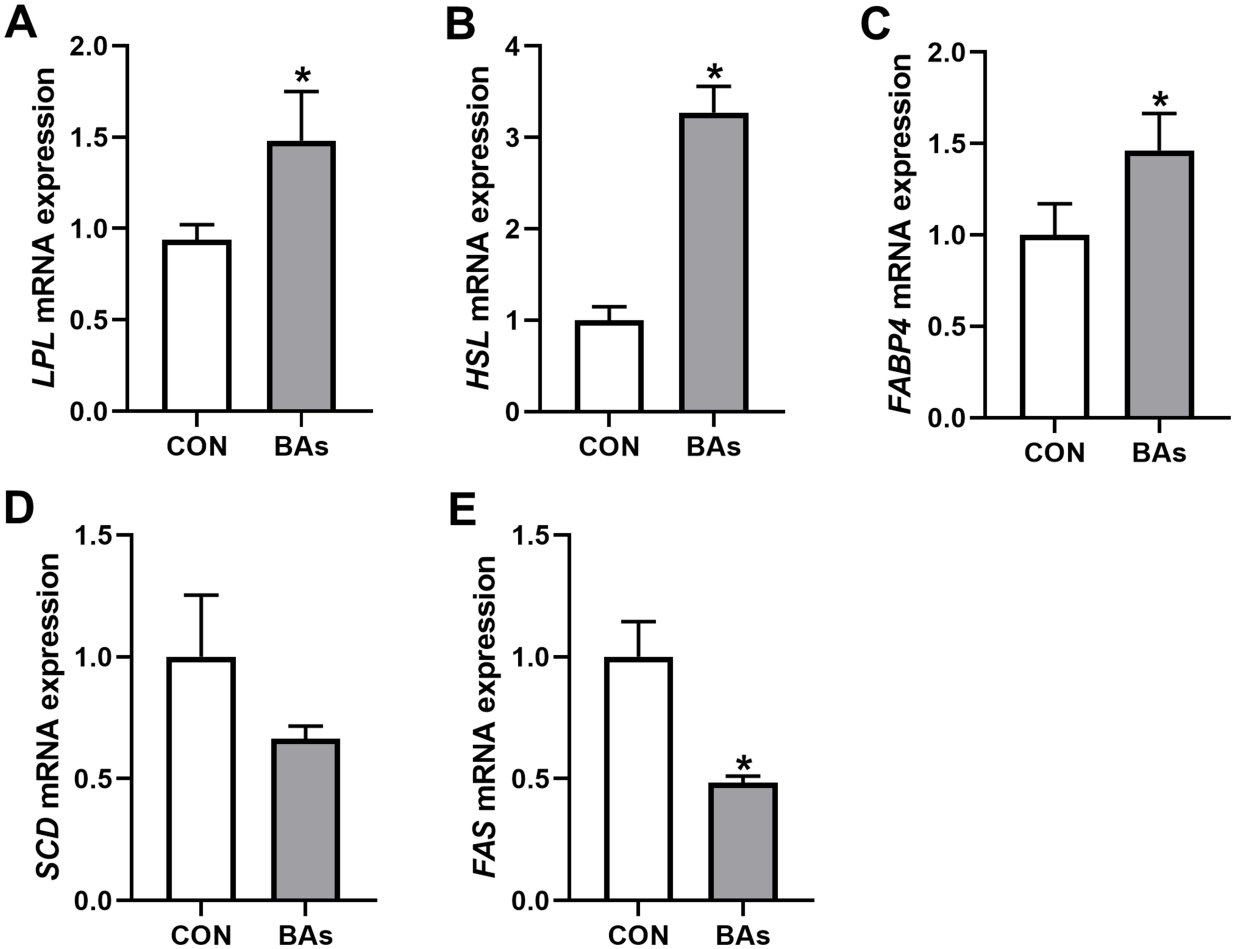
Effects of BAs supplementation on liver lipid metabolism in the broilers. **(A–E)** mRNA expression of lipoprotein lipase (LPL) **(A)**, hormone-sensitive lipase (HSL) **(B)**, fatty acid-binding protein 4 (FABP4) **(C)**, stearoyl coenzyme A desaturase (SCD) **(D)**, and fatty acid synthase (FAS) **(E)**. The results were expressed as means ± SEMs, *n* = 8. *p-value < 0.05 compared to the CON group.

To further evaluate the effects of BAs on liver lipid metabolism in broilers, the mRNA expressions of lipid metabolism genes such as *LPL*, *HSL*, *FABP4*, *SCD*, and *FAS* were determined. As shown in Figure 4, the mRNA expressions of *LPL*, *HSL*, and *FABP4* were significantly increased by 57.9, 215.6, and 46.3%, respectively (*p* < 0.05), while the mRNA expression of *FAS* was significantly decreased by 51.6% in the BAs group compared to the CON group (*p* < 0.05). However, the relative mRNA expression of *SCD* did not change significantly between the two treatments (*p* > 0.05).

### 3.5 Liver morphology examination

**Figure 5:**
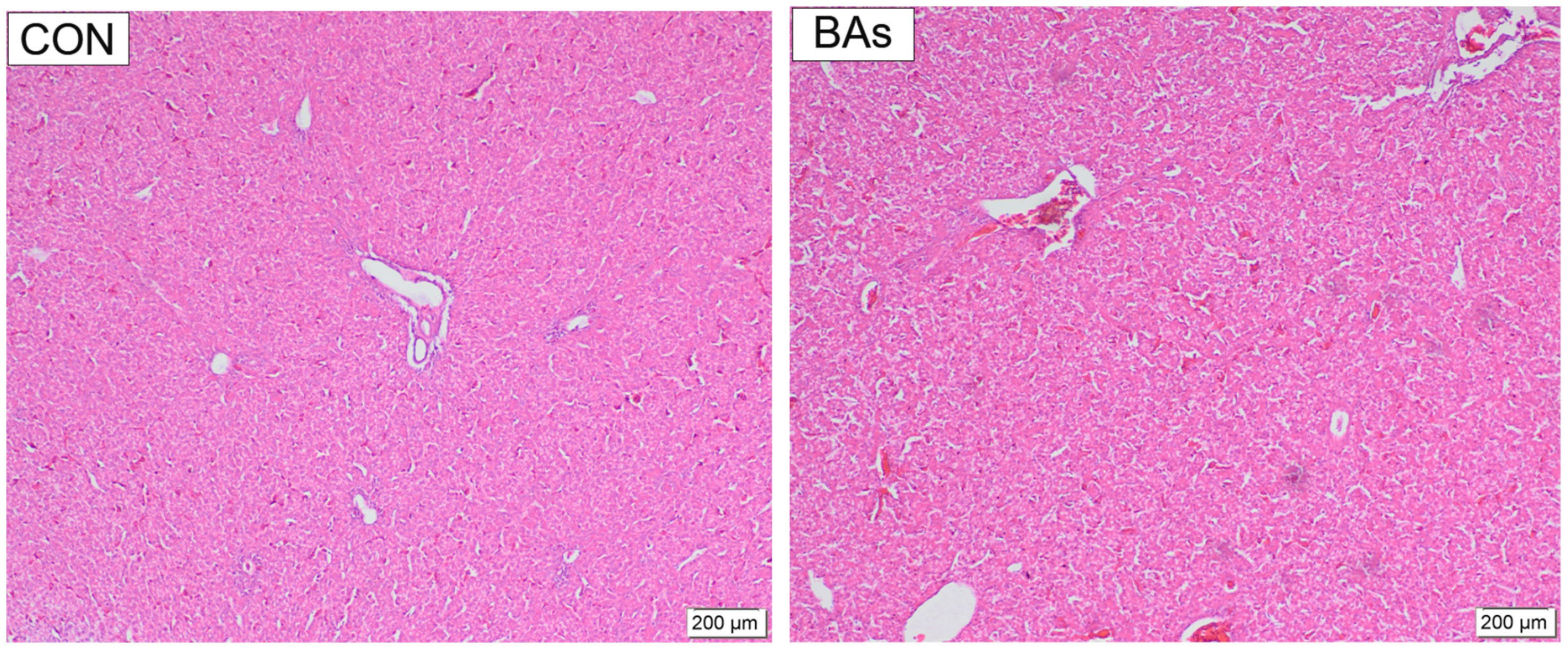
Effects of BAs supplementation on liver morphology in the broilers. The scale bars indicate 200 μm, *n* = 8.

The effects of BAs on liver morphology in broilers are shown in Figure 5. Hepatocytes were well-developed with clear boundaries, and the central vein structure of the hepatic lobules was intact and dilated in both treatments. Partial blood cell infiltration was observed in the veins and tissues of the CON group, which was alleviated by the addition of BAs. Compared to the CON group, the nuclei of the BAs group showed darker staining, increased size, a polygonal shape, enlarged hepatic blood sinusoids, and clear hepatic cell cords.

### 3.6 Intestinal morphology examination

**Figure 6:**
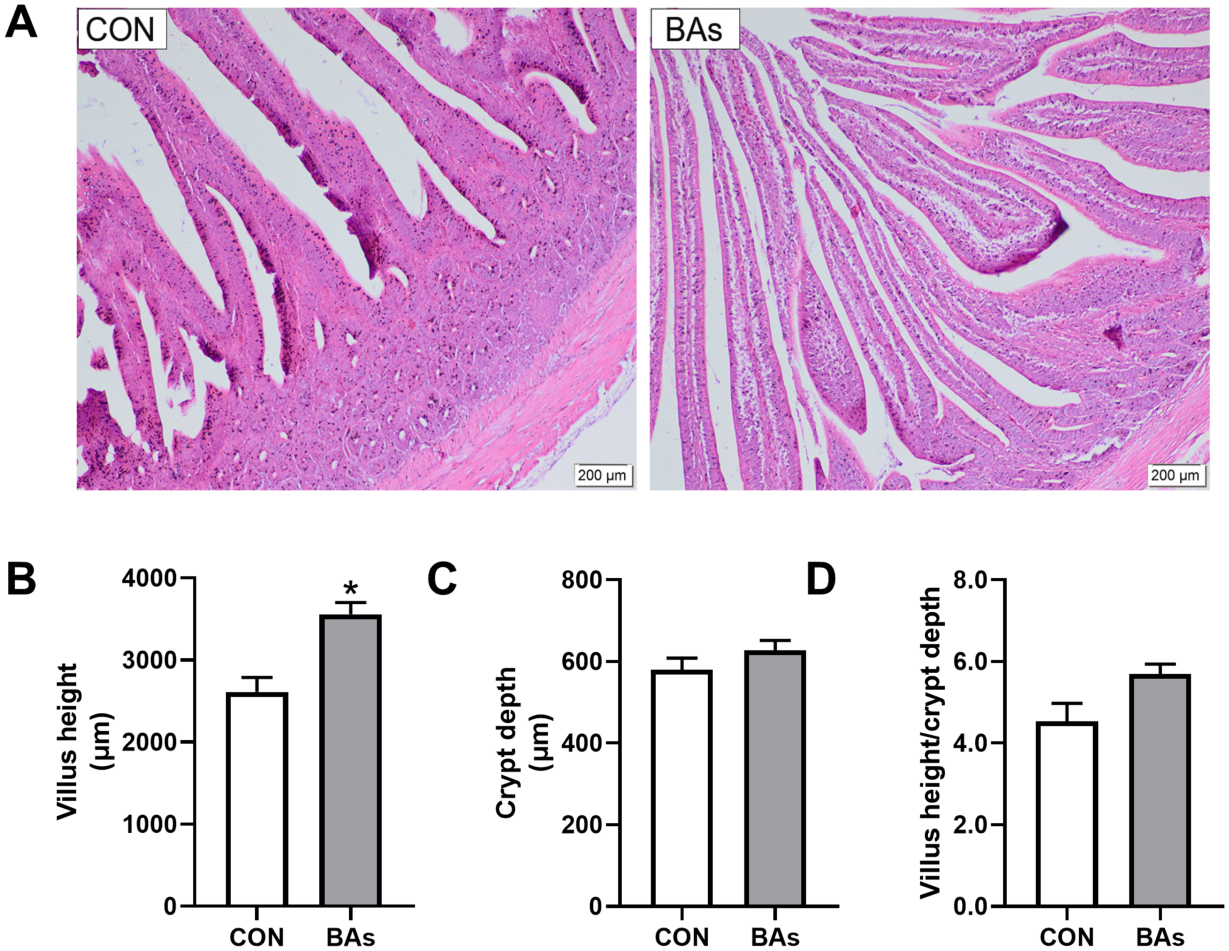
Effects of BAs supplementation on jejunal morphology in the broilers. **(A)** Representative hematoxylin and eosin staining images. **(B)** VH, villus height. **(C)** CD, crypt depth. **(D)** VH/CD ratio, villus height-to-crypt depth ratio. The results were expressed as means ± SEMs, *n* = 8. The scale bars indicate 200 μm. *p-value < 0.05 compared to the CON group.

The effects of BAs on jejunal morphology in broilers are shown in Figure 6. Compared to the CON group, BAs significantly increased villus height by 36.3% (*p* < 0.05) but had no significant effect on crypt depth (*p* > 0.05), with a tendency to increase the villus height/ crypt depth ratio (0.05 < *p* < 0.1). Meanwhile, the addition of BAs improved jejunal morphology, as evidenced by clear and shiny villi, thickening, lengthening, a mostly intact mucosal epithelium, occasional epithelial abscission, and an increase in the number of villi in the same field of view.

## 4 Discussion

### 4.1 Production performance

In broiler diet formulation, fats and oils are frequently used as the most direct energy sources, helping to raise dietary energy density while also improving feed texture and palatability (17, 18). However, poor digestion and absorption of dietary lipids can lead to excessive accumulation of fat in livestock. Abdominal fat tissue is the main site of lipid storage, and excessive abdominal fat deposition could adversely affect feed efficiency and carcass quality in broilers (19, 20). Emulsifiers are a class of nutritive feed additives that promote fat digestion and absorption. It has been reported that the addition of exogenous emulsifiers to diets containing moderate amounts of added vegetable fat may significantly improve broiler performance (21). Furthermore, other studies have also reported inefficient digestion and absorption of fat in young broilers due to low levels of natural endogenous lipase production. Supplementation with exogenous emulsifiers has shown beneficial effects on growth performance and may also affect meat color and lipid metabolism (22). BAs are a class of amphipathic steroids produced through the catabolism of cholesterol in the liver. They promote the emulsification of lipids and enhance lipid digestion and utilization in livestock (23, 24). Therefore, BAs are commonly used as exogenous emulsifiers in broiler diets to improve meat quality and enhance slaughter performance. Zhang et al. (25) found that BA supplementation significantly reduced body fat and tail fat weight in lambs. Consistently, the present study found that BA supplementation significantly decreased abdominal fat yield in the broilers. Similarly, Chen et al. (26) found that the abdominal fat ratio of broilers supplemented with BAs showed a decreasing trend, while the quality of breast muscle mass significantly improved. However, the present study did not observe any significant effect of BAs on muscle, which is presumed to be due to the influence of BAs concentrations supplied in the diets.

### 4.2 Serum biochemistry

Serum biochemical indicators can partially reflect an organism’s metabolic and health status (27, 28). Serum TG, HDL, and LDL are biochemical indicators closely related to lipid metabolism (29). Lai et al. (13) observed that dietary supplementation with BAs at doses of 40, 60, and 80 mg/kg had no significant effects on serum TG, HDL, and LDL levels in broilers, which was consistent with the findings of the present study. ALT and AST are the two enzymes with the highest transaminase activity in animals and are primarily found in liver and myocardial cells, with minimal serum levels (30). When tissue cells are damaged or the permeability is increased, ALT and AST are released into the bloodstream, leading to elevated serum ALT and AST activity (31). The activities of AST and ALT in serum are commonly used as clinical indicators for diagnosing liver injury (32). Our research showed that BAs supplementation significantly reduced serum AST activity, which was consistent with previous findings (33). This suggests that BAs were able to alleviate liver damage to some extent by altering serum parameters.

### 4.3 Liver morphology and lipid metabolism

The liver is the most important metabolic and detoxification organ in the body, and the structural integrity of liver cell membranes is responsible for the exchange of substances between liver cells and the bloodstream (34). It was reported that hepatocytes in the BA group had normal morphology, with intact cell membranes and well-defined borders (35), which was consistent with the current study’s finding that BAs could improve liver health. Lipid metabolism primarily occurs in the liver and directly affects fat deposition in broilers (36). A previous study showed that BAs play a crucial role as signaling molecules in lipid metabolism (37). FAS was reported to promote fat accumulation and catalyze the synthesis of fatty acids (38). It was also reported that BAs treatment significantly inhibited the activity of FAS, resulting in a reduction in lipid synthesis (39), which was consistent with the results of this study. However, LPL, a tissue-specific enzyme, is primarily expressed in the adipose tissue and liver of young animals, playing a crucial role in lipid degradation (40). In addition, HSL and FABP4 are highly expressed in adipocytes and could promote lipolysis (41, 42). It has been shown that the addition of emulsifiers significantly increases the mRNA expression levels of LPL in the liver of broilers (8), which was consistent with the current study. This study also showed that BAs significantly increased the mRNA expressions of HSL and FABP4 in the liver of the broilers. Therefore, the results suggest that exogenous BAs benefit liver health by down-regulating the mRNA expression of lipogenesis-related genes and up-regulating the expression of lipolysis-related genes.

### 4.4 Jejunal morphology

Intestinal morphology is closely related to animal growth (43). The VH, CD, and VH/CD ratios of intestinal epithelial cells reflect the functional status and nutrient absorption capacity of the intestine (44, 45). Studies have reported that BAs play an essential role in the emulsification and absorption of dietary fat and vitamins in the small intestine and could promote intestine growth and development (46, 47). This study showed that BAs supplementation significantly increased jejunal villus height and improved jejunal morphology in the broilers. Consistent with this, BAs also increased duodenal villus height and the VH/CD ratio, thereby enhancing the nutrient absorption capacity of the intestine (48). Another study showed that BAs supplementation significantly increased the villus height of the ileum in broilers, strengthening the intestinal mucosal barrier (49). Therefore, BAs could improve intestinal morphology and promote intestinal health.

## 5 Conclusion

In summary, dietary supplementation with BAs in broilers can effectively improve carcass characteristics and serum enzyme activity, regulate lipid metabolism, and improve liver and intestinal morphology, thereby promoting liver and intestinal health. Based on current research, BAs are optional additives for improving broiler health.

## Data Availability

The datasets presented in this study can be found in online repositories. The names of the repository/repositories and accession number(s) can be found in the article/Supplementary material.
