# Supplementary material for: Effects of bile acids on production performance, serum biochemistry, lipid metabolism, and intestinal morphology in broilers
Source: Front Vet Sci. 2025 Apr 30;12:1584544. doi: 10.3389/fvets.2025.1584544 (PMC12075112; doi:10.3389/fvets.2025.1584544)
Supplement: Supplementary file 1 [file Table_1.DOCX]

Supplementary Material

# Supplementary Figures and Tables

## Supplementary Table

**Table S1.** Primer sequences for quantitative real-time PCR

| **Gene^2^** | **GenBank** | **Primer Sequence, 5’ to 3’^1^** | **Size, bp** |
| --- | --- | --- | --- |
| *HSL* | XM_040657096.1 | F: CCATCCTGTCCGTCGATTACTC | 80 |
|  |  | R: GCAGTAGGCGTAGAAGCACTC |  |
| SCD | NM_204890.2 | F: TCCACAACTACCACCATACATTCC | 97 |
|  |  | R: AGGCACATGAGGTCGATGAAG |  |
| *LPL* | NM_205282.2 | F: CAGTGCAACTTCAACCATACCA | 150 |
|  |  | R: AACCAGCCAGTCCACAACAA |  |
| *FAS* | NM_001199487.2 | F: TTGTTCGTCATCACCGTCTATCG | 136 |
|  |  | R: GTTTCGTAGGCTCCTCCCATTC |  |
| *FABP4* | NM_204290.2 | F: TGATGAGACCACAGCAGATGAC | 120 |
|  |  | R: TCCACCACTTTCCTCTTGATAACA |  |
| *β-Actin* | NM_001170517.2 | F: CCACGAAACTACCTTCAACTC | 131 |
|  |  | R: TGATCTCCTTCTGCATCCTGT |  |

^1^ F: forward prime; R: reverse primer. ^2^ HSL, hormone sensitive lipase; SCD, stearoyl coenzyme a desaturase; LPL, lipoprotein lipolytic enzyme; FAS, fatty acid synthase; FABP4, fatty acid binding protein 4.
